# Association between abnormal body mass index and pregnancy outcomes in patients following frozen embryo transfer: a systematic review and meta-analysis

**DOI:** 10.1186/s12958-021-00809-x

**Published:** 2021-09-09

**Authors:** Jiaqi Yang, Yichen He, Yiqing Wu, Dan Zhang, Hefeng Huang

**Affiliations:** 1 Key Laboratory of Reproductive Genetics (Ministry of Education) and Department of Reproductive Endocrinology, Zhejiang University, School of Medicine, Women’s Hospital, Xueshi Road, No.1, Hangzhou, 310006 Zhejiang province China; 2 International Peace Maternal and Child Health Hospital, School of Medicine, Shanghai Jiao Tong University, No. 910, Rd. Hengshan, Shanghai, 200030 China

**Keywords:** FET, BMI, Live birth rate, ART, Meta-analysis

## Abstract

**Background:**

There has been increasing interest in the relationship between body mass index(BMI) and pregnancy outcomes, especially in women undergoing frozen embryo transfer(FET). Several observational studies have been published, but so far with conflicting results.

**Methods:**

A systematic review and meta-analysis was conducted according to PRISMA guidelines. Pubmed, Embase, Cochrane Library, Clinicaltrails.gov and Web of Science databases were searched based on established search strategy from inception through January 2021.

**Results:**

Twelve studies were eligible. In women following FET, high BMI (BMI ≥ 23 kg/m^2^) was associated with an impaired live birth rate (LBR, OR: 0.89, 95% CI: 0.82–0.96, *P* = 0.002), but wasn’t associated with the implantation rate or the clinical pregnancy rate. Subgroup analysis revealed higher LBR for women didn’t complicated by polycystic ovary syndrome (PCOS, OR: 0.96, 95% CI: 0.85–1.08, *P* = 0.46) and women with blastocyst transferred (OR: 0.89, 95% CI: 0.68–1.16, *P* = 0.40). LBR did not differ between the low BMI group (BMI < 18.5 kg/m^2^) and the normal weight group.

**Conclusions:**

Our study showed that high BMI in women is negatively associated with LBR in FET cycles, whereas low BMI isn’t. The results of subgroup analysis implied a need for women with a high BMI to get individualized weight management and treatment. Further evidence is still required to optimize preconception health and develop Nutritional and exercise guidelines.

## Background

There has been increasing interest in the relationship between body mass index(BMI) and reproductive outcomes [1–3]. The adverse effects of overweight/obesity on pregnancy outcomes have been widely confirmed, including dysregulation of the hypothalamic-pituitary-ovarian axis, ovulation disorders, impaired preimplantation embryo, and higher risk of miscarriage, stillbirth, and preeclampsia [4]. As in patients who undergo assisted reproduction technology(ART), elevated BMI may lead to higher doses of gonadotropins, higher risks of ovarian hyperstimulation syndrome and miscarriage, increased cancellation rates, and lower oocyte recovery [5, 6]. Though it is still on debating [7, 8], underweight women may have higher rates of anovulatory and lower fecundity [9, 10]. During in vitro fertilization (IVF) cycles, the relationship between patients with a low BMI and IVF outcomes turn out to be more inconsistent, most previous studies are limited by small sample sizes [7, 9, 11–13].

Compared with fresh cycles, frozen embryo transfer (FET) allows the timing of transfers more flexible, and the embryos into a more physiologic uterine environment, have drawn much attention in recent years [14, 15]. An increasing number of observational studies and a meta-analysis which investigated the relationship between IVF outcomes and female obesity, have suggested a decreased probability of live birth in obese (BMI ≥ 30 kg/m^2^) women compared with women with a normal weight (BMI 18.5–24.9 kg/m^2^) [16]. However, almost all records included in the meta-analysis were based on fresh embryo transfers, and the underweight group was not included. Given the quite different treatment and the maternal status between fresh and frozen cycles, the effect of abnormal BMI on FET outcomes deserves a separate assessment. Several observational studies evaluating the effect of abnormal BMI on pregnancy outcomes have been published, but thus far, conflicting results have been reported.

We therefore conducted a systematic review incorporating all the published studies and included a meta-analysis to evaluate the association between high BMI and pregnancy outcomes, including live birth rate (LBR), implantation rate and clinical pregnancy rate following FET. Subgroups analyses were performed according to embryo stage, ovarian status, BMI category and cycle rank. The relationship between female underweight and LBR was also studied.

## Methods

Our review was conducted followed by the PRISMA guidelines for systematic reviews and meta-analyses [17]. A review protocol was registered in the international prospective register of systematic reviews PROSPERO (ID CRD42021232400).

### Search strategy

The Pubmed(MEDLINE), Embase, Cochrane Library, Clinicaltrails.gov and Web of Science databases were searched with no time restrictions for relevant literature. Only studies published in English or Chinese were included. Key search terms will be the following the text words: ((“Embryo Transfer”[Mesh/Emtree] And “Frozen”) OR (“Embryo Transfer”[Mesh/Emtree] And “Frozen-thawed”) OR (“Embryo Transfer”[Mesh/Emtree] And “cryopreservation”) OR “FET” OR “Frozen embryo transfer” OR “frozen-thawed embryo transfer” OR (“Blastocyst Transfer” And “Frozen”), OR (“Blastocyst Transfer” AND “Frozen-thawed”) OR (“Blastocyst Transfer” And “cryopreservation”)) AND (“Body Mass Index” OR “Obesity” OR “obese” OR “Overweight”) AND (“Pregnancy Outcome” OR “Live Birth” OR “Pregnancy Outcome” OR “obstetric outcome” OR “perinatal outcome” OR “Reproductive outcomes”).

### Eligibility criteria and quality assessment

According to the National Institute of Health (NIH) and the World Health Organization (WHO), an abnormal BMI was identified as a BMI ≥ 25 kg/m^2^ or BMI ≤ 18.5 kg/m^2^ [18]. However, latter evidence suggested that Asian populations may have a high risk of type 2 diabetes and cardiovascular disease in the existing WHO BMI category and therefore require a lower BMI cut-off points to determine overweight and obesity [19]. In certain countries, the BMI cut-off points are more concrete. Therefore, the existing literature has shown considerable heterogeneity on BMI category. To be considered for inclusion, all observational studies (cohort studies and case report studies) assessed the relationship between abnormal BMI and FET outcomes were included. As compensation for inconsistency, the original BMI cut-off points and mean ± SD value of BMI in each group were noted for further subgroup analyses. Studies are required to report values of live birth for BMI, if one study described implantation rate or clinical pregnancy rate for BMI either, the data would also be noted.

In study selection and quality assessment stage, two reviewers (J.Q.Y. and Y.C.H) independently performed a screening of titles and abstracts of all searched studies, and relevant full-text articles were further assessed based on the inclusion criteria to evaluate the risk of bias. Any discrepancies or uncertainties were resolved by consensus with a third reviewer (Y.Q.W).

The risk of uncontrolled bias in the studies will be assessed using the Newcastle–Ottawa Scale(NOS) [20], each study was judged by three perspectives: study selection (inclusion–exclusion criteria, population), comparability between groups (age and embryo quality, studies that provided greater control of confounding factors such as cause of infertility, endometrial preparation protocol, endometrial thickness, number of transferred embryos and PCOS scored with additional stars) and evaluation of the outcome and follow-up. The NOS criteria and scoring system were fully described. Quality was ranked as low (0–5 points), intermediate (6–7 points), or high (8–9 points). Only studies with a score of more than 5 points were included. Publication bias assessment was performed with funnel plots.

### Data extraction and statistical analysis

We generated a descriptive table for population and study characteristics about all eligible studies, including the first author, publication year, country, study design, BMI category, mean ± SD value of BMI, inclusion–exclusion criteria, embryo state of transferred, ovarian status, cycle rank and endometrial preparation protocol. For each group (normal weight, high or low BMI), the sample size, and the number of live births were noted, if the original data was record as a percentage of live birth, they were transferred into a number of live births according to the sample size.

Statistical analysis was carried out using the software Review Manager 5.3.5 (Copenhagen: The Nordic Cochrane Centre, The Cochrane Collaboration, 2014). Meta-analysis was performed using a random effects model with the Mantel–Haenszel (M-H) method. The I^2^ statistic was used to assess the impact of heterogeneity across the studies, I^2^ ≥ 50% indicated substantial heterogeneity [21]. The magnitude of the effect of will be estimated by calculating the odds ratio (OR) with 95% confidence interval (CI). Pooled effect sizes were deemed statistically significant at *P* < 0.05.

## Results

### Study selection and study characteristics

**Fig. 1:**
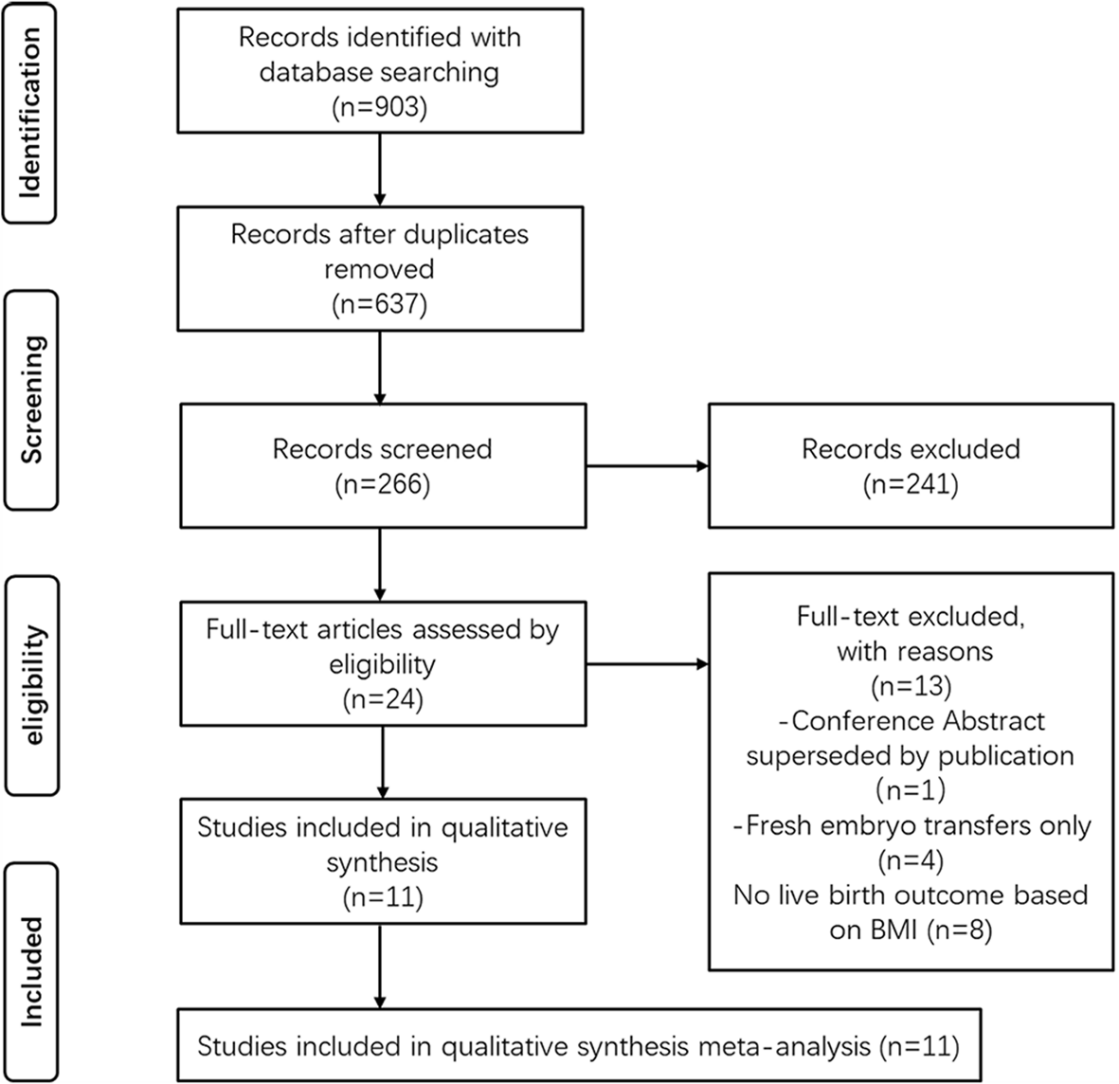
The flow chart of study selection for the systematic review

A flow diagram of study identification for the meta-analysis is shown in Fig. 1. The search strategy identified a total of 903 articles, after removing duplicates, 266 abstracts were further reviewed, and irrelevant articles were excluded. 25 full-text articles were assessed for eligibility and quantitative analysis. Among them, four articles explored the association between BMI and reproductive outcomes with fresh embryo transfers only, eight were excluded for no live birth outcomes based on BMI, and one article was a conference abstract superseded by publication. All 12 studies had data available for BMI and for correlated live birth, which seemed potentially appropriate for inclusion in the meta-analysis.

In aggregate, eleven of eligible studies had information about high BMI and live births, including 42,724 FET cycles [22–32], and seven studies considered underweight women, including 34,300 FET cycles [23, 24, 26, 28, 29, 32, 33]. Most were conducted in autologous cycles [22, 24–32], only one study taken donor cycles into consideration [23]. Participants were recruited mainly from China [22, 24, 25, 28, 29, 31, 32], the USA [23, 33], the UK [30], France [27], and Turkey [26]. Studies considering embryo transfer stage, ovarian status, and cycle rank are presented in Table 1. Given that there are only a handful of different methods for preparing the uterine endometrium and that all included studies confirmed the thickness of endometrium on the day of embryo transferred reached a certain value (7 or 8 mm), we believe these studies were of similar methodological quality.

**Table 1:** Characteristics of studies included in the meta-analysis

Study	Country	Year	Definition of normal BMI	Low BMI	Normal weight	High BMI	Patients	Embryo stage	Autologous or donor oocytes cycle	Ovarian status of the patients	Cycle rank	Endometrial preparation
Chen et al. [22]	China	2018	18.5–24	NA	21.1 ± 1.5	26.8 ± 2.3	Inclusion: Age ≤ 35 years, with PCOS, First IVF/ICSI cycles, GnRH-ant protocol for COH Exclusion: Congenital adrenal hyperplasia, androgen secreting tumors, or Cushing’s syndrome	Both	Autologous	Only PCOS	All	depends on patients' condition
Insogna et al. [23]	USA	2017	18.5–24.9	Not specified	Not specified	Not specified	Inclusion: All consecutive FBT cycles Exclusion: Incomplete information cycles, cancelled cycles	Blastocyst	Both	Both	All	HRT after pituitary suppression
Jin et al. [24]	China	2019	18.5–23.9	Not specified	Not specified	Not specified	Inclusion: Age < 35 years; tubal factors or primary infertility; poor semen quality; retro grade ejaculation or obstructive azoospermia for ICSI; normal menstrual cycle Exclusion: PCOS; endometriosis; endocrine abnormalities; chromosome polymorphism or chromosome abnormalities; adverse pregnant production history; ovarian dysfunction; ovarian surgery or uterine malformation; donated oocytes	Both	Autologous	Not PCOS	First	depends on patients' condition
Lin et al. [25]	China	2019	18.5–24.9	NA	21.45 ± 1.7	28.6 ± 2.1	Inclusion: PCOS; Age 20–35 years; first FET cycles Exclusion: History of unilateral oophorectomy; abnormalities of the uterus; karyotypic abnormalities; recurrent pregnancy loss; any conditions which precluded the safety of pregnancies or ART	Both	Autologous	Only PCOS	First	HRT or mild stimulation
Oliva et al. [33]	USA	2020	18.5–24.9	Not specified	Not specified	NA	Inclusion: Age 20–46 years, all patients with a documented BMI Exclusion: BMI ≥ 25 kg/m^2^	Blastocyst	Autologous	Not specified	Not specified	HRT
Ozgur et al. [26]	Turkey	2019	18.5–24.9	Not specified	Not specified	Not specified	Inclusion: Age 18–42 years, First FET, ICSI cycles Exclusion: Not specified, only single blastocyst transfers were analyzed in live birth rate	Blastocyst	Autologous	Not specified	First	HRT after pituitary suppression
Prost et al. [27]	France	2020	18.5–24.9	NA	21.5 ± 1.8	34 ± 3.1	Inclusion: All consecutive autologous FBT cycles Exclusion: Oocyte donation, natural or stimulated, PGT cycles; risk factors for recurrent pregnancy loss	Blastocyst	Autologous	Both	All	HRT
Qiu et al. [28]	China	2019	18.5–24.9	17.62 ± 0.8	21.86 ± 1.8	28.1 ± 1.6	Inclusion: PCOS Exclusion: Serious and unstable disease; gynecological borderline and malignant tumors; other metabolic disorders; chromosomal abnormalities; congenital uterine malformations	Both	Autologous	Only PCOS	First	HRT or mild stimulation
Tang et al. [29]	China	2021	18.5–24.9	17.53 ± 3.1	21.61 ± 1.7	26.37 ± 1.9	Inclusion: Age 20–40 years, FSH < 10 IU/L Exclusion: Lack of antral follicles or FSH > 10 IU/L; diabetes or hypertension; recurrent pregnancy loss; endocrine abnormalities; drugs or diseases that can cause underweight	Both	Autologous	Not PCOS	First	depends on patients
Rittenberg et al. [30]	UK	2011	18.5–24.9	NA	21.6 ± 1.9	27.8 ± 2.2	Inclusion: Single blastocyst transfer Exclusion: Age > 40 years, BMI < 18.5 kg/m^2^; PGD, donated oocytes; embryos frozen for fertility preservation; monozygotic twin gestation	Blastocyst	Autologous	Both	Not specified	HRT after pituitary suppression
Wang et al. [31]	China	2017	18.5–24.9	NA	21.28 ± 0.0	27.13 ± 0.1	Inclusion: Age < 40 years; FSH < 10 IU/L; AFC of more than 3; regular menstrual cycles; male, unexplained, or tubal factors infertility Exclusion: Accept HRT treatments in previous 3 month; PCOS; functional ovarian cyst with E2 > 100 pg/mL; PGD, IVM, donor cycles; any contraindications to ovarian stimulation treatment; presence of fresh embryo transplantation	Both	Autologous	Not PCOS	Not specified	depends on patients' condition
Zhang et al. [32]	China	2019	18.5–22.9	17.66 ± 0.7	20.67 ± 1.2	25.68 ± 1.5	Inclusion: First FET cycles, high quality embryo Exclusion: Age > 40 years; recurrent pregnancy loss; previous IVF attempts; submucosal fibroids or polyps, intramural fibroids > 4 cm, hydrosalpinx, and congenital uterine malformation; hypertension; diabetes; thyroid dysfunction	Both	Autologous	Both	First	depends on patients' condition

Note: *BMI* body mass index, *E2* estradiol, *PCOS* polycystic ovary syndrome, *COH* controlled ovarian hyperstimulation, *ART* assisted reproductive technology, *FET* frozen embryo transfer, *FBT* frozen blastocyst transfer, *GnRH* gonadotropin-releasing hormone, *FSH* follicle-stimulating hormone, *ICSI* intracytoplasmic sperm injection, *IVF* in vitro fertilization, *AFC* antral follicle count, *NA* not applicable, *PGD* Preimplantation Genetic Diagnosis, *PGT* Preimplantation Genetic Testing, *IVM* in vitro maturation, *HRT* Hormone replacement therap

Most studies met the four standard WHO categories for BMI(underweight, normal weight, overweight and obese were defined based on a respective BMI < 18.5 kg/m^2^, ≥ 18.5 BMI < 24.9 kg/m^2^, ≥ 25 kg/m^2^, and BMI ≥ 30 kg/m^2^) [23, 25–31, 33]; one study used the Asian BMI classification [32], namely, normal weight was 18.5–22.9 kg/m^2^ [18], and two studies stratified patients according to the Chinese standard [22, 24], and defined normal weight as 18.5–24 kg/m^2^. Since eligible studies outlined the BMI classification differently and to delimit a homogenous definition among the included studies in the meta-analysis, we set 18.5 kg/m^2^ ≤ BMI ≤ 22.9 kg/m^2^ for normal BMI and pooled all of the predefined overweight and obese patients in which BMI sets were more than 23 kg/m^2^ for high BMI group. To ensure that participants in studies with higher BMI cut-off points (BMI between 23 and 24.9) were not mistakenly assigned to normal weight group, we noted mean ± SD value of BMI in each study. Eight studies had available data and showed mean value of control group ranged from 20.67–21.82 kg/m^2^, and the overall heterogeneity was moderate at 40%, which could be tolerated.

### Primary outcome: association between LBR and high BMI

#### Overall LBR outcomes

**Fig. 2:**
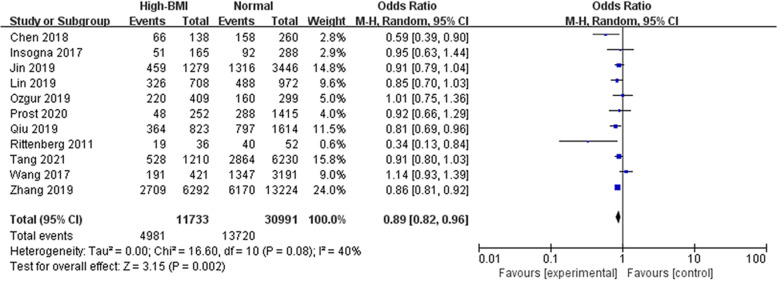
Overall effect of high BMI on the live birth rate. ‘Events’ relates to FET cycles leading to live birth, and ‘Total’ relates to the total number of FET cycles included in the study. A high BMI was considered BMI ≥ 23 kg/m^2^, and a normal weight was considered BMI 18.5–22.9 kg/m^2^

**Fig. 3:**
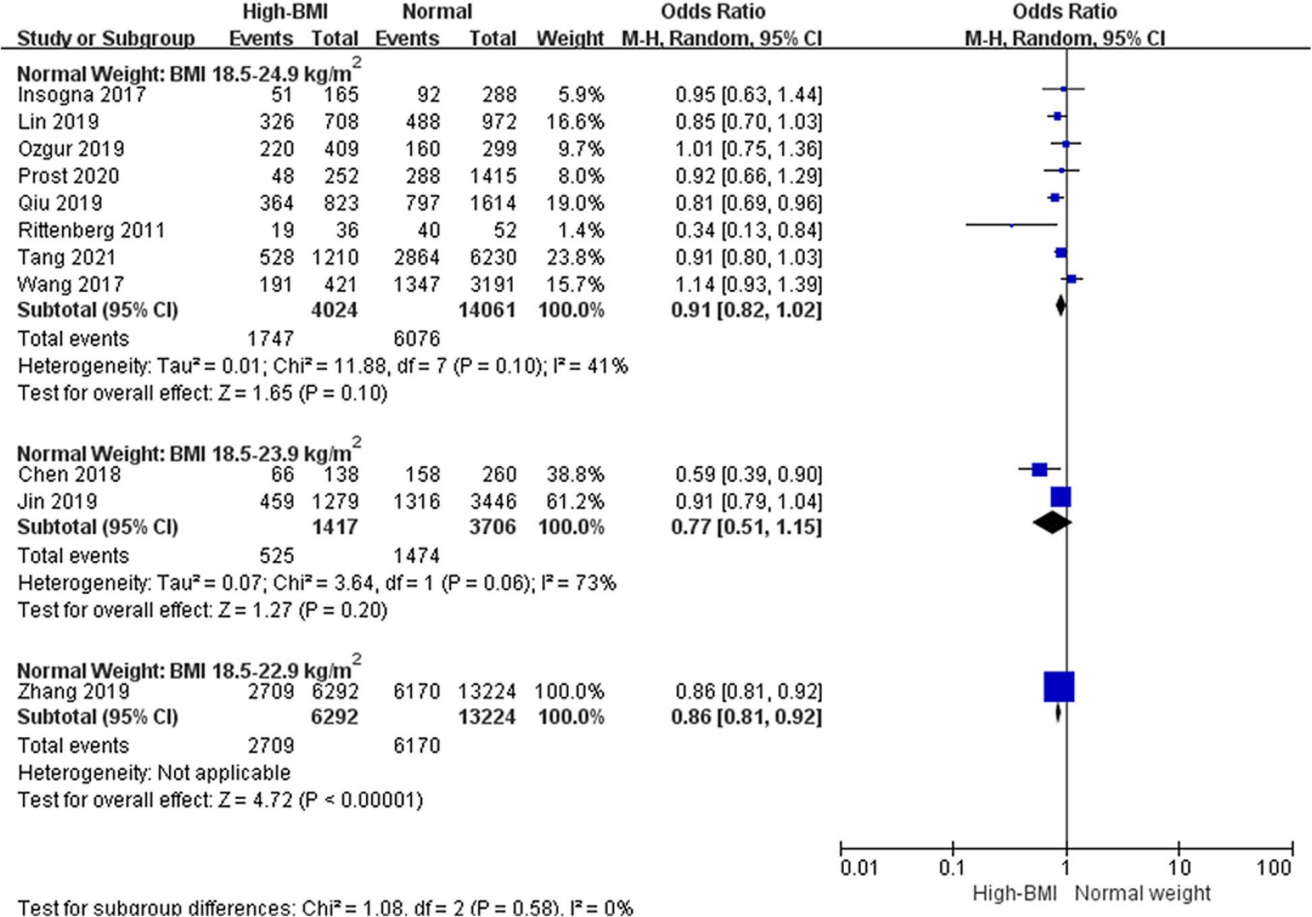
Subgroup analysis according to BMI category. ‘Events’ relates to FET cycles leading to live birth, and ‘Total’ relates to the total number of FET cycles included in the study

From the meta-analysis, high-BMI overall (BMI ≥ 23 kg/m^2^) has significantly adverse effect on live birth (OR: 0.89, 95% CI: 0.82–0.96, *P* = 0.002, I^2^ 40%) compared with a BMI in the normal range (Fig. 2). Subgroup analyses were further conducted according to BMI standards (Fig. 3), it turns out that there was no association between high BMI and live birth when the cut-off point was 25 kg/m^2^ (OR: 0.91, 95% CI: 0.82–1.02, *P* = 0.10, I^2^ = 41%).

#### Subgroup analyses for LBR

**Fig. 4:**
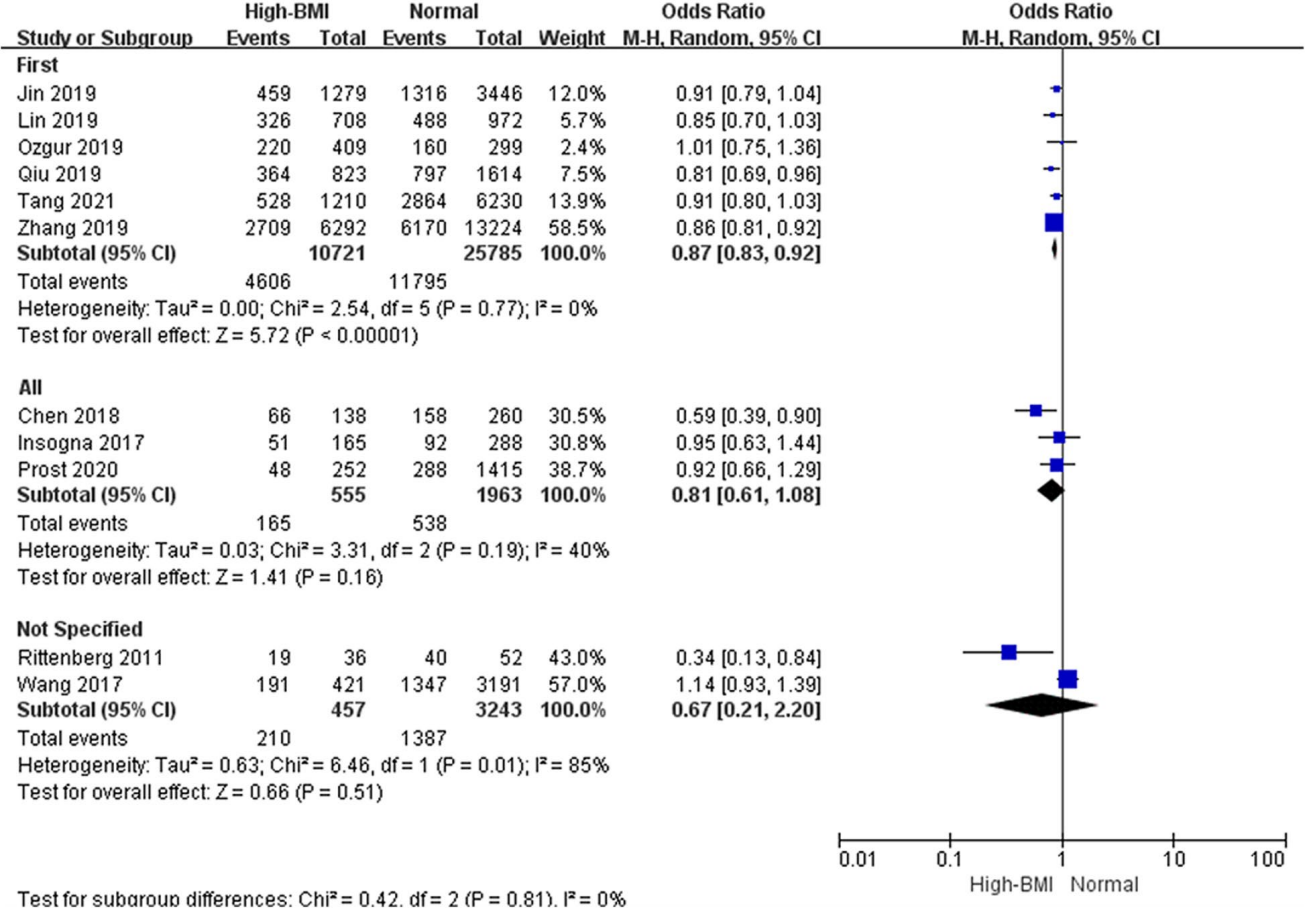
Subgroup analysis according to cycle rank. ‘Events’ relates to FET cycles leading to live birth, and ‘Total’ relates to the total number of FET cycles included in the study

**Fig. 5:**
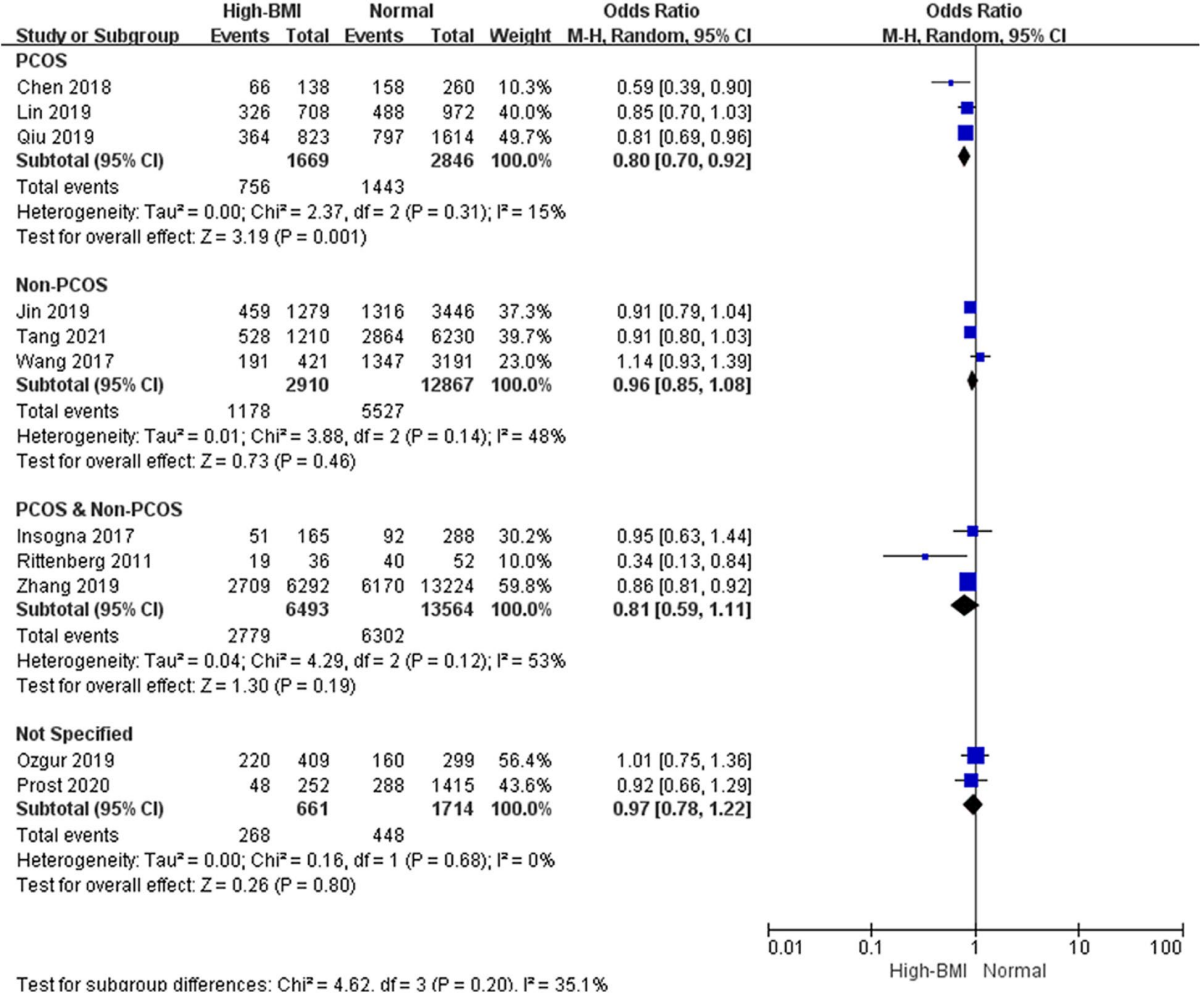
Subgroup analysis according to ovarian status. ‘Events’ relates to FET cycles leading to live birth, and ‘Total’ relates to the total number of FET cycles included in the study

**Fig. 6:**
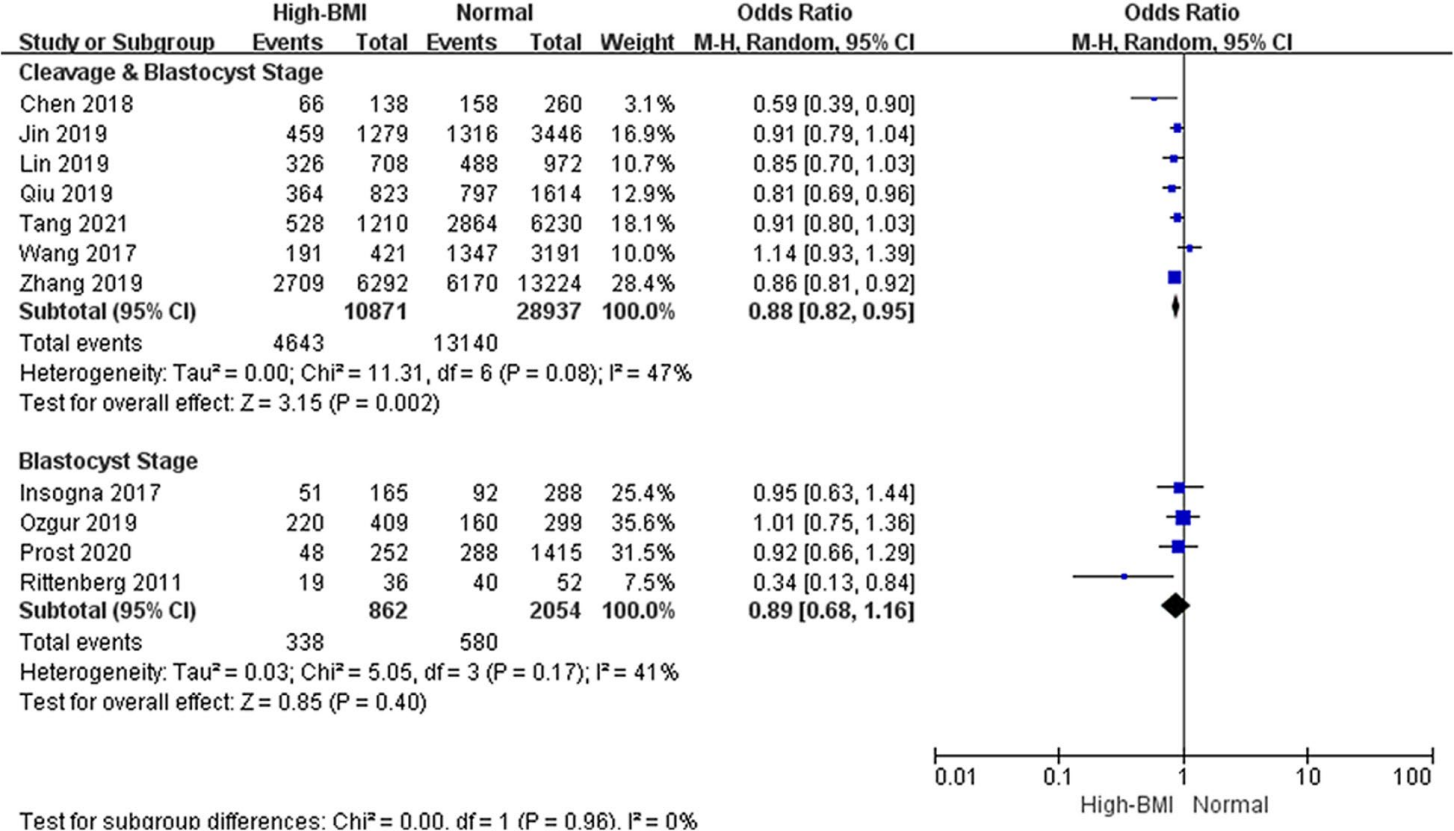
Subgroup analysis according to embryo stage. ‘Events’ relates to FET cycles leading to live birth, and ‘Total’ relates to the total number of FET cycles included in the study

Subgroup analyses were performed according to cycle rank (first, all, not specified, Fig. 4), indicating that a high BMI adversely affected LBR in the first cycle of FET but not in all cycles. Studies considering the first FET cycle combined analysis with a total of 36,506 cycles showed good homogeneity and significantly lower LBR in women with a high BMI than in women with a normal weight (OR: 0.87, 95% CI: 0.83–0.92, *P* < 0.001, I^2^ = 0%), whereas LBR was comparable between obese women and women with a normal weight when all FET cycles were considered. We also performed subgroup analyses according to ovarian status (PCOS, non-PCOS, PCOS & non-PCOS, not specified, Fig. 5). Pooled data from three studies considering PCOS patients, suggested a lower LBR in PCOS women than in women with a normal weight (OR: 0.80, 95% CI: 0.70–0.92, P = 0.001, I^2^ = 15%). However, the same interpretation was not observed in studies that selected only women without PCOS (OR: 0.96, 95% CI: 0.85–1.08, *P* = 0.46, I^2^ = 48%), and three eligible studies showed a mediation effect (OR: 0.81, 95% CI: 0.59–1.11, *P* = 0.19, I^2^ = 53%). It seemed that women with PCOS were more vulnerable to the adverse effect of high BMI on live birth than those without PCOS. Subgroup analyses was performed according to embryo stage (cleavage & blastocyst, blastocyst, Fig. 6). Four studies reported on only blastocyst transferred showed that the negative association between high BMI and LBR might be modified (OR: 0.89, 95% CI: 0.68–1.16, *P* = 0.40, I^2^ = 41%).

### Secondary outcomes

#### Implantation rate and Clinical pregnancy rate associated with high BMI

**Fig. 7:**
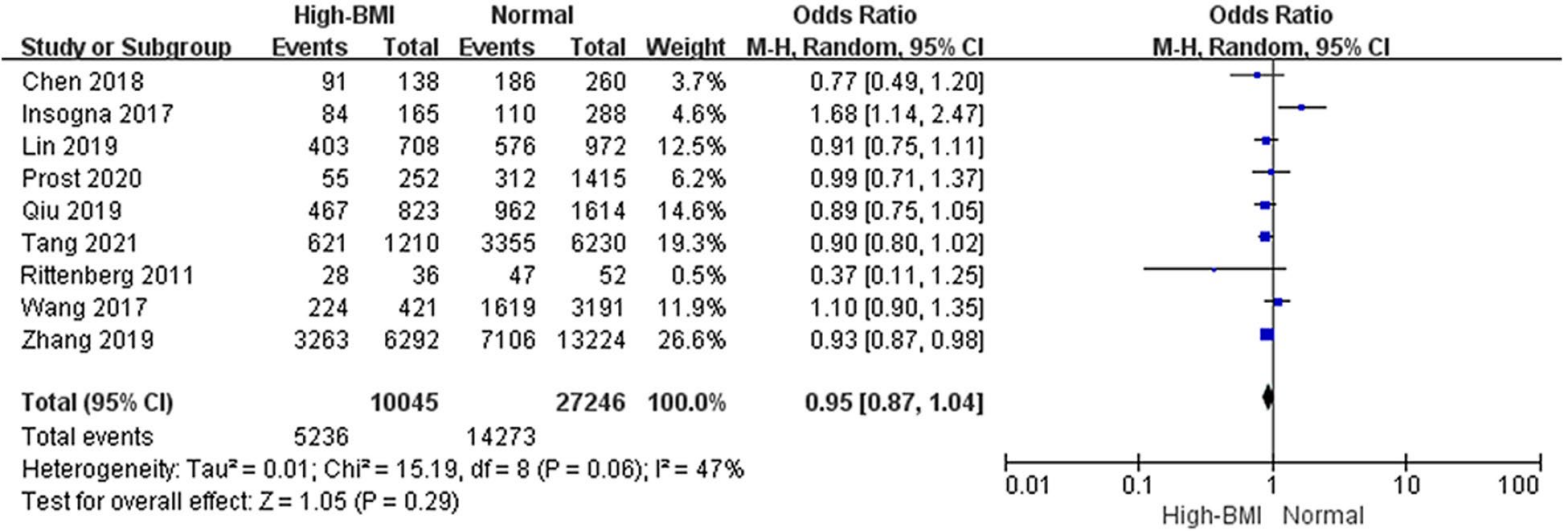
Effect of high BMI on clinical pregnancy rate. ‘Events’ relates to FET cycles leading to clinical pregnancy, and ‘Total’ relates to the total number of FET cycles included in the study

**Fig. 8:**
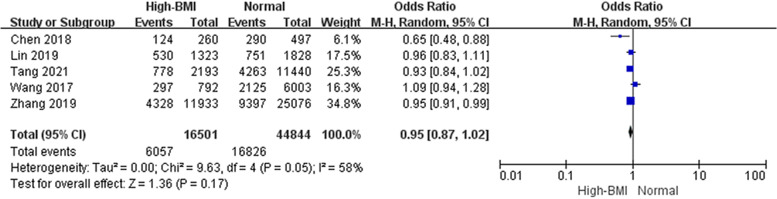
Effect of high BMI on implantation rate. ‘Events’ relates to FET leading to implantation, and ‘Total’ relates to the total number of embryos transferred included in the study

When it comes to early pregnancy, nine studies analyzed 37,291 cycles showed no difference in the clinical pregnancy rate between high BMI and women with a normal weight (Fig. 7, OR: 0.95, 95% CI: 0.87–1.04, *P* = 0.29, I^2^ = 47%). Furthermore, there was no difference in the implantation rate across five studies including 61,345 embryo transferred (Fig. 8, OR: 0.95, 95% CI: 0.87–1.02, *P* = 0.17, I^2^ = 58%).

#### Association between LBR and low BMI

**Fig. 9:**
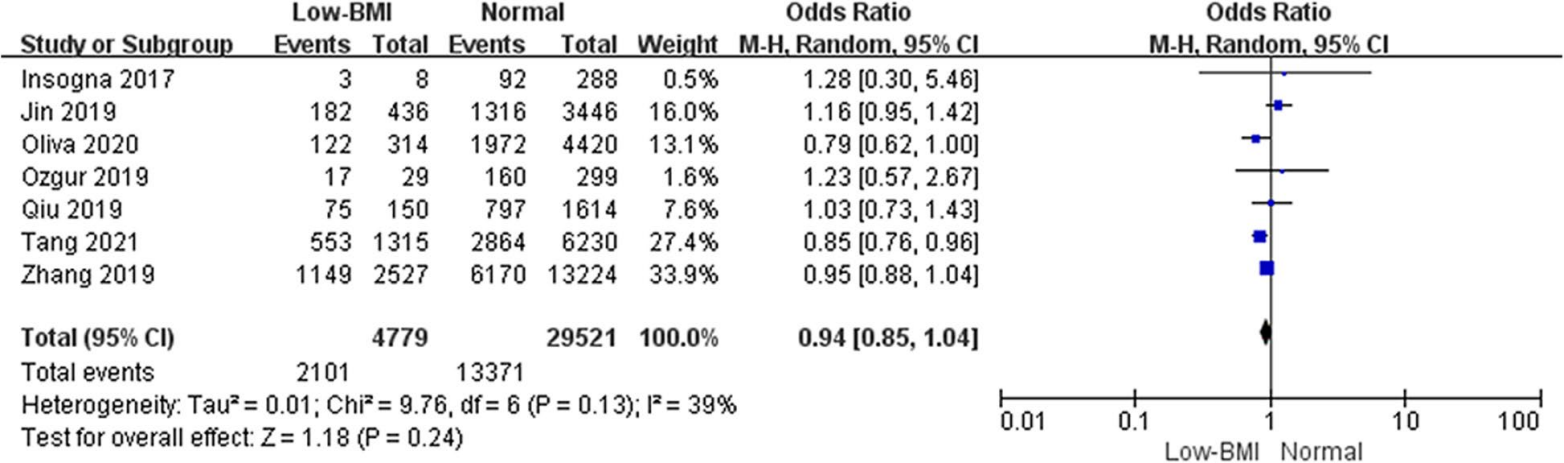
Live birth rate following FET in Low BMI and normal weight women. A low BMI was considered BMI < 18.5 kg/m^2^, and a normal weight was considered BMI 18.5–22.9 kg/m^2^

In addition, we conducted a meta-analysis to evaluate the effect of underweight (BMI < 18.5 kg/m^2^) on live birth. There was no difference in LBR between underweight women compared with women with a normal weight (Fig. 9, OR: 0.94, 95% CI: 0.85–1.04, *P* = 0.24, I^2^ = 39%).

### Quality assessment

#### Risk of bias

**Fig. 10:**
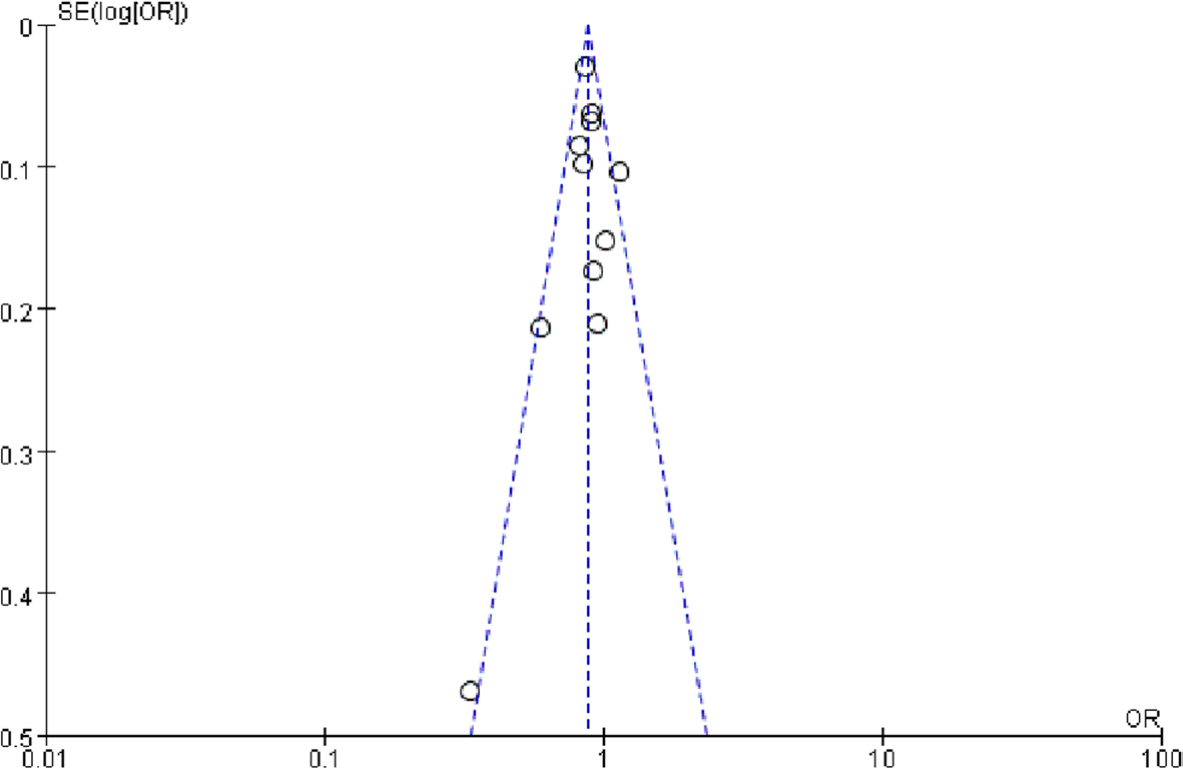
Funnel plot analysis for assessing publication bias

We employed the Newcastle–Ottawa scale for quality assessment of the studies that included in the meta-analysis, and the scoring system is provided in Table 2. Overall, the quality assessment of these studies showed a low risk of bias. Among the nine applicable stars assessing the participants selection, comparability and outcomes, the eligible studies received six to nine stars. And funnel plot analysis showed no obvious publication bias (Fig. 10).

**Table 2:** The Newcastle–Ottawa Scale(NOS) scores of the studies included in the meta-analysis

	Selection				Comparability	Outcome			
Study	Representativeness of the exposed cohort	Selection of the non-exposed cohort	Ascertainment of exposure	Demonstration that outcome of interest was not present at start of study	Control for important factors ^**a**^	Assessment of outcome	Was follow-up long enough for outcomes to occur	Adequacy of follow-up of cohorts	Scores
Chen et al. [22]	-	★	-	★	★	★	★	★	6
Insogna et al. [23]	★	★	★	★	★★	★	★	★	9
Jin et al. [24]	-	★	★	★	-	★	★	★	6
Lin et al. [25]	-	★	★	★	★★	★	★	★	8
Oliva et al. [33]	★	★	★	★	-	★	★	★	7
Ozgur et al. [26]	★	★	-	★	★	★	★	★	7
Prost et al. [27]	★	★	★	★	★★	★	★	★	9
Qiu et al. [28]	-	★	★	★	★	★	★	★	7
Tang et al. [29]	★	★	★	★	★	★	★	★	8
Rittenberg et al. [30]	★	★	★	★	★	★	★	★	8
Wang et al. [31]	★	★	★	★	★	★	★	★	8
Zhang et al. [32]	★	★	★	★	★★	★	★	★	9

^a^ A maximum of 2 stars can be allotted in this category, one for age, the other for other controlled factors

#### Sensitivity analyses

**Fig. 11:**
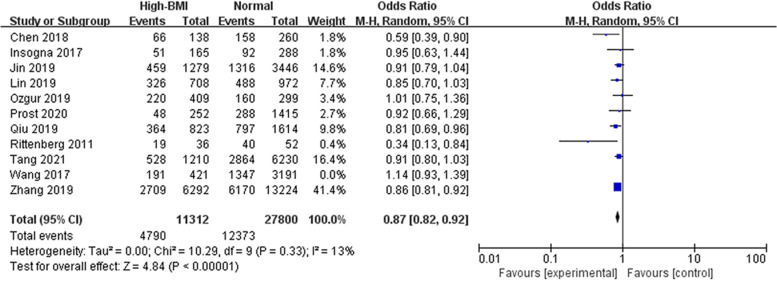
Effect of high BMI on the live birth rate when excluded the outlier study. ‘Events’ relates to FET cycles leading to live birth, and ‘Total’ relates to the total number of FET cycles included in the study

We used a fixed effects model and did not modify the overall result (0.88, 0.84–0.86) (data not shown). Sensitivity analyses was conducted by excluding eligible studies one at a time, and one study was revealed to be an outlier [31]. The results were not influenced when the data from Wang et al. was excluded. OR (95% CI) for a live birth following FET was 0.86 (0.81–0.92) in women with a high BMI when compared to women with a normal weight, with a pretty low heterogeneity (Fig. 11).

## Discussion

In our review, data from 12 studies demonstrates that high BMI didn’t impact early pregnancy proxy such as implantation rate and clinical pregnancy rate but associated with decreased LBR following FET. Additionally, women with a low BMI didn’t show the same effect. Thus, our study mainly confirmed that women with high BMI had impaired outcomes in FET cycles. This result has to be interpreted carefully however, especially because one included study provided almost half of the data, which may skew the results. FET are believed to enable maternal embryos to enter a more physiological condition than fresh embryos [34, 35]. Our research compensated the earlier vacancy, found that even in FET cycles the adverse effect can not be reversed.

Considering the complexity of reproductive process, which components are affected most by a high BMI are largely unknown. Since our study was based on frozen cycles, and all cycles had at least one selected embryo transferred, the hypothesis that a high BMI may affect LBR by damaging oocyte maturation and reducing the number of retrieved oocytes was not applicable. However, a high BMI is still believed to influence oocyte metabolism and quality by altering composition of the follicular fluid [1, 36, 37] and damaging mitochondrial function in the oocyte [38], thus lead to increased risk of embryo aneuploidy and poor quality embryos [39, 40]. In addition, data from diet-induced obesity mouse models showed that a high BMI impaired following reproductive processes such as embryonic development [37, 41, 42] and the preimplantation stage [43]. Whereas evidence from donor oocyte cycles found no association between recipient with a high BMI (BMI ≥ 25 kg/m^2^) and IVF outcomes [44], which suggested that oocyte quality rather than others is the overriding factor influencing IVF outcomes in obese women using autologous oocytes. Our results considering about the implantation rate and clinical pregnancy rate tended to support the assumption that high BMI didn’t impact the preimplantation stage or early embryonic development. Alternatively, FET treatment could rescue the effects of high BMI in this period.

PCOS, a series of metabolic disorders, is associated with subfertility [45–48]. It’s been reported however, patients with PCOS undergone FET could have promising pregnancy outcomes rather than fresh embryo transfers [49]. Due to limitations in our study design, we couldn’t investigate when PCOS complicated by high BMI, whether FET can modify the overall effect compared with fresh cycles. Yet our results confirm that PCOS patients are more sensitive to the effect of high BMI thus have a poorer FET outcome than non-PCOS patients, which implied that women with PCOS might require a stricter weight management than those without PCOS.

Following our established research strategy, we didn’t find studies reported only cleavage-stage embryo transfers with documented BMI, but four studies included blastocyst transfers. Although this result is based on only 2916 cycles, women with a high and BMI blastocyst-stage embryo transfer had a higher LBR than those regardless of embryo stage, which supports the preceding research result [50]. Despite there would be loss in the process of blastocyst culture, the financial and emotional burdens of failure could be more intolerable. Therefore, it might be better for women with a high BMI to get blastocyst transfer rather than cleavage embryo transfer.

Earlier theory showed a U-shaped association between a high or low BMI and pregnancy outcomes after IVF [12, 51]. In our study, we failed to show that a low BMI could cause disparities in LBR. This is in accordance with some studies that women with a low BMI have similar IVF and pregnancy outcomes to those with a normal BMI [7, 13, 23, 52, 53]. Combined with the interpretation of high BMI, our results provide reassurance to underweight patients undergoing FET, which would give a better guide to optimize preconception weight.

Our study has some limitations. First, we identified high BMI as BMI ≥ 23 kg/m^2^ rather than using the definition of overweight/obesity according to the WHO standardized classification of BMI. The noted mean value of normal weight group ranged from 20.67–21.82 kg/m^2^, which means that these participants are basically satisfied our criteria. However, it still presents relatively heterogeneity in terms of BMI definitions. Second, as LBR was the main outcome, we evaluated the implantation rate and clinical pregnancy rate, but failed to assess additional outcomes. However, as LBR is the gold standard reproductive outcome, one result was mainly concerning that homogeneity can be guaranteed. Third, even if we sought to control for the quality of the included studies carefully, some confounding parameters such as ovarian stimulation protocols, endometrium preparation, and embryo quality, might still have unintentionally introduced bias into our study results. Our meta-analysis has several strengths. To our knowledge, no prior meta-analysis performed a separate assessment of the relationship between abnormal BMI and FET outcomes. Our results are helpful to provide individualized weight management advice for women undergoing FET, and shed new light on the effect of underweight on live birth.

## Conclusions

In conclusion, our meta-analysis demonstrates that high BMI in women is negatively associated with LBR even in FET cycles, whereas low BMI isn’t. Complication with PCOS may induce patients to be more vulnerable to the detriment impact of high BMI, and it might be a better idea for women with a high BMI to receive blastocyst transfer. This information might be helpful for women and their providers to individualize weight management and treatment, however, nutritional and exercise guidelines for optimizing preconception health are still encouraged to be further discussed.

## Data Availability

All data are available in this paper.

## Abbreviations

OR: Odds ratio
CI: Confidence interval
BMI: Body mass index
ART: Assisted reproductive technology
IVF: In vitro fertilization
FET: Frozen embryo transfer
LBR: Live birth rate
NIH: National Institute of Health
WHO: World Health Organization
NOS: Newcastle–Ottawa Scale
PCOS: Polycystic ovary syndrome
PRISMA: Preferred Reporting Items for Systematic Reviews and Meta-Analysis
